# Afucosylated anti-EBOV antibody MIL77-3 engages sGP to elicit NK cytotoxicity

**DOI:** 10.1128/jvi.00685-24

**Published:** 2024-08-20

**Authors:** Yuting Zhang, Min Zhang, Haiyan Wu, Xiaonan Wu, Hang Zheng, Junjuan Feng, Mianjing Wang, Jing Wang, Longlong Luo, He Xiao, Chunxia Qiao, Xinying Li, Yuanqiang Zheng, Weijin Huang, Youchun Wang, Yi Wang, Jiannan Feng, Guojiang Chen

**Affiliations:** 1 Institute of Pharmacology and Toxicology, Beijing, China; 2 Joint National Laboratory for Antibody Drug Engineering, Henan University, Kaifeng, China; 3 Inner Mongolia Key Lab of Molecular Biology, School of Basic Medical Sciences, Inner Mongolia Medical University, Hohhot, China; 4 Division of HIV/AIDS and Sex-transmitted Virus Vaccines, National Institutes for Food and Drug Control, Beijing, China; 5 Department of Hematology, The Fifth Medical Center, Chinese PLA General Hospital, Beijing, China; The Ohio State University, Columbus, Ohio, USA

**Keywords:** ebola virus, antibody function, natural killer cells, ADCC

## Abstract

MIL77-3 is one component of antibody cocktail that is produced in our lab and represents an effective regimen for animals suffering from Zaire Ebolavirus (EBOV) infection. MIL77-3 is engineered to increase its affinity for the FcγRIIIa (CD16a) by deleting the fucose in the framework region. The potential effects of this modification on host immune responses, however, remain largely unknown. Herein, we demonstrated that MIL77-3 recognized secreted glycoproptein (sGP), produced by EBOV, and formed the immunocomplex to potently augment antibody-dependent cytotoxicity of human peripheral blood-derived natural killer cells (pNKs), including CD56^dim^ and CD56^bright^ subpopulations, in contrast to the counterparts (Mab114, rEBOV548, fucosylated MIL77-3). Intriguingly, this effect was not observed when NK92-CD16a cell line was utilized and restored by the addition of beads-coupled or membrane-anchored sGP in combination with MIL77-3. Furthermore, sGP bound to unrecognized receptors on T cells contaminated in pNKs rather than NK92-CD16a cells. Administration of beads-coupled sGP/MIL77-3 complex in mice elicited NK activation. Overall, this work reveals an immune-stimulating function of sGP/MIL77-3 complex by triggering cytotoxic activity of NK cells, highlighting the necessity to evaluate the potential impact of MIL77-3 on host immune reaction in clinical trials.

**IMPORTANCE:**

Zaire Ebolavirus (EBOV) is highly lethal and causes sporadic outbreaks. The passive administration of monoclonal antibodies (mAbs) represents a promising treatment regimen against EBOV. Mounting evidence has shown that the efficacy of a subset of therapeutic mAbs *in vivo* is intimately associated with its capacity to trigger NK activity, supporting glycomodification of Fc region of anti-EBOV mAbs as a putative strategy to enhance Fc-mediated immune effector function as well as protection *in vivo*. Our work here uncovers the potential harmful influence of this modification on host immune responses, especially for mAbs with cross-reactivity to secreted glycoproptein (sGP) (e.g., MIL77-3), and highlights it is necessary to evaluate the NK-stimulating activity of a fucosylated mAb engaged with sGP when a new candidate is developed.

## INTRODUCTION

Zaire Ebolavirus (EBOV) and related viruses of the family Filoviridae are highly lethal and have caused numerous outbreaks since emerging in 1967, including the largest epidemic in West Africa from 2013 to 2016 (1). The passive administration of monoclonal antibodies (mAbs) emerged as a promising treatment approach against EBOV (2). To date, several mAbs and mAb cocktails have entered clinical development, and two of them (REGN-EB3, Mab114) were approved by FDA (3). ZMapp contains three antibodies—mAbs 4G7 and 2G4 from ZMab and mAb 13C6 from MB-003 directed against EBOV glycoprotein (GP), which were produced in Nicotiana tobacco plant cells with afucosylated and/or agalatosylated glycans (4, 5). Attempts to manufacture ZMapp on a large scale met with challenges due, in part, to low 4G7 yields in both plant and mammalian expression systems. To overcome this obstacle, we developed a new version of the cocktail called MIL77, which was composed of MIL77-1 (containing the variable regions of 2G4), MIL77-2 (containing the variable regions of 4G7), and MIL77-3 (containing the variable regions of 13C6) (6). The antibodies in MIL77 have modified framework regions to be more similar to human counterparts. The CHO cells used for MIL77 expression are engineered to prevent fucosylation similar to the *N*-glycosylation present in plant-produced ZMapp. The absence of fucose increases the affinity of the mAbs for the FcγRIIIa (CD16a) as well as Fc-mediated immune functions (7). Furthermore, we demonstrated that a two-antibody cocktail, including MIL77-1 and MIL77-3, provided full protection in nonhuman primates after a challenge with a lethal dose of EBOV (6). Although enhanced Fc-mediated NK activation might contribute to the efficacy of the cocktail treatment, the potential effects of this modification on host immune responses remain poorly understood.

The EBOV GP is the sole viral envelope protein and a major component directed by therapeutic mAbs. It is encoded by the GP gene along with two truncated versions: soluble GP (sGP) and small soluble GP (ssGP) as a result of transcriptional editing (8). GP and sGP share 295 N-terminal amino acids but have distinct C-terminal regions. In fact, sGP is the primary product of the GP gene and is secreted in abundance during EBOV infection (9, 10). Antibodies elicited during infection that cross-react to sGP and GP can potentially be absorbed by sGP, suggesting that sGP is important for virus survival within the host (11, 12).

In this study, we demonstrated that therapeutic mAbs (MIL77-3, Mab114, rEBOV548) directed against GP also recognized sGP. The immunocomplex of sGP and MIL77-3, instead of Mab114 and rEBOV548, had the potency to induce pNK activation and cytokine release. This effect was tightly associated with Fc glycoengineering of MIL77-3 and crosslinking of sGP to the receptors on immune cells.

## MATERIALS AND METHODS

### Cell culture assays

Peripheral blood mononuclear cells (PBMCs) were separated from the peripheral blood of healthy donors within 4 h of collection using Ficoll–Hypaque centrifugation according to the manufacturer’s instructions. pNKs were then enriched by negative selection using the NK cell isolation kit (Miltenyi Biotec, Cat. No. 130092657) with the purity of over 95% (CD45^+^CD3^−^CD56^+^) (Fig. S1) and expanded for 2 weeks in the presence of human IL-2 (200 U/mL, Sino Biological, Cat. No. 11848-HNAE2). NK92-CD16a were purchased from the Chinese Academy of Sciences and cultured in 37℃, 5% CO_2_ and maintained in α-MEM (Gibco, Cat. No. C12571500BT) supplemented with 12.5% fetal bovine serum (Gibco, Cat. No. 10099141C), 12.5% horse serum (Gibco, Cat. No. 26050088), 100 µM 2-mercaptoethanol (Thermo, Cat. No. 21985023), 100 U/mL penicillin, and 500 U/mL IL-2 streptomycin (Gibco, Cat. No. 15140122).

### Protein or antibody expression and purification

EBOV GP (Uniprot: Q05320, aa33-632, ∆312–464), sGP (Uniprot: P60170), and ssGP (Uniprot: Q9YMG2) with six-histidine-tag at C-terminus were cloned into pcDNA3.1 vectors for expression in HEK293T cells. The proteins were purified by nickel column (GE Healthcare, Cat. No. 11003399) and stored at −80 °C.

CHO-S cells were purchased from American Type Culture Collection (ATCC) and cultured in ExpiCHO Expression Medium (Gibco, Cat. No. A2910001) in an orbital shaker incubator (Kuhner) at 37°C, 8% CO_2_. MIL77-3 plasmid was transfected into the glyco-engineered CHOK1-AF cells for expression (6). The CHOK1-AF cell line was developed by engineering the CHO-K1 (CCL-61, ATCC) using the zinc-finger nuclease technology to knock out the SLC35C1 gene, which encodes the GDP-fucose transporter, a critical factor to regulate the fucosylation of glycans (6, 13). MIL77-3F, Mab114, rEBOV548, MIL77-1, ADI-15946, rEBOV520 plasmids were transfected into CHO-S according to the manufacturer’s instructions. Purification was performed using the ÄKTAprime Plus system (GE Healthcare). The Biotin Labeling Kit (Elabscience, Cat. No. ELKB002) was used to label mAbs and sGP according to the instructions.

#### Preparation of F(ab’)_2_

Pepsin (Sigma, Cat. No. 107185) was diluted with 20 mM sodium acetate (pH 4.0). Pepsin was mixed with antibody (1:8) and incubated at 37°C for 6 h. The reactions were stopped with the addition of 2 M Tris-base solution to yield a solution with pH 7.5. The cleaved antibody was mixed with protein A beads (TRAN, Cat. No. DP30101) and rotated at room temperature for 30 min to enable full binding of the beads to the Fc. The effluent F(ab’)_2_ was collected and ultrafiltered with a 30 kDa centrifugal (Millipore). SDS-PAGE was performed to identify the purity of recombinant proteins and mAbs.

### SPR analysis

The SPR (surface plasmon resonance) analysis was performed using a Biacore T200 machine with CM5 chips (GE Healthcare) at room temperature. All the proteins used in SPR analysis were exchanged to BIAcore buffer (HBS-EP containing 0.03 M EDTA and 0.5% Surfactant P20, pH 7.4). The chip was subsequently immobilized with sGP protein in sodium acetate (pH 5.0) and then blocked with 1 M ethanolamine (pH 8.0). Ligands (MIL77-3, Mab114, rEBOV548) were diluted by running buffer at the concentrations ranging from 6.25 to 400 µM. The chip was regenerated with glycine-HCl (10 mM pH 2.0). Data were analyzed by Biacore T200 Evaluation Software.

### ELISA

#### Indirect ELISA

sGP (2 µg/mL) was coated in carbonate buffer (pH 9.6) at 4°C overnight. The plate was sealed with 4% nonfat milk at 37°C for 1 h to block non-specific binding. Mabs (MIL77-3, MIL77-3F, Mab114, rEBOV548, MIL77-1, ADI-15946, rEBOV520) were threefold serially diluted from 5 µg/mL in a total of 9 dilution concentrations. The reaction was carried out at 37°C for 1 h, and the unconjugated product was washed. Horseradish peroxidase (HRP)-conjugated goat anti-human IgG (H + L) secondary antibody (Thermo, Cat. No. 31130) was added and incubated at 37°C for 30 min. Binding signals were visualized using a TMB substrate (CWBIO, Cat. No. CW0050), and the reaction was stopped by adding 2 N H_2_SO_4_. Absorbance at 450 nm was measured by an enzyme-labeled instrument.

#### Competitive ELISA

sGP (2 µg/mL) was coated overnight. Biotinylated-MIL77-3 (0.5 µg/mL), -mAb114 (0.1 µg/mL), or -rEBOV548 (0.8 µg/mL) was used to dilute MIL77-3, mAb114, or rEBOV548 in threefold serial dilution (ranging from 0.823 to 200 µg/mL). The biotin-mAbs and mAbs complex were then added to the plates. After 1 h incubation at 37°C, the plates were washed and the bound biotin-mAbs were detected by adding HRP-labeled streptavidin (Thermo, Cat. No. S911). The remaining steps were the same as above.

### Analysis of IgG glycan

The *N*-glycan analysis of the MIL77-3, MIL77-3F, rEBOV548, and mAb114 was determined by the H-Class-Q-Tof LC-MS system. First, the samples of mAbs were desalted with purified water by ultrafiltration, and then PNGase F (NEB, Cat. No. P0705L) was added and incubated with antibodies in 37°C water-bath for 16 h to release oligosaccharides. After enzyme digestion, the oligosaccharides, which were later separated by −20℃ frozen absolute ethanol, along with standard GU Ladder glycan (Merck, Cat. No. 31417), were placed in a vacuum concentrator for drying. 2-AB (Sigma, Cat. No. A89804) and the reducing agent sodium cyanoborohydride were used to obtain labeling reagent. Then, the dried samples were naturally cooled to room temperature and mixed with labeling reagent, incubated in 65℃ metal-bath at dark for 3 h. After samples were labeled, Mobile phase A (50 mM ammonium formate aqueous solution, pH 4.4) and Mobile phase B (pure acetonitrile) were added, vortexed, and centrifugated at 12,000 rpm for 5 min. The supernatants of mixed solutions were then loaded onto the Glycan BEH Amide chromatographic column (Waters). Data analysis was performed by ChemStation.

### Lentivirus preparation and infection

For lentivirus constructs, sGP-CD8α, CAR19 ScFv (control)-CD8α transmembrane (TM) region, full-length GP sequences, and lentiviral control plasmid were inserted into pHBLV-CMV-MCS-EF1-ZsGreen lentiviral vectors (Hanbio), respectively. The recombinant lentivirus was produced by co‐transfection of HEK293T cells with the plasmids pHBLV-CMV-MCS-EF1-ZsGreen, psPAX2, and pMD2G with LipoFiter (Hanbio, Shanghai, China). Lentivirus‐containing supernatants were harvested 48 h after transduction and filtered through 0.22‐μm cellulose acetate filters (Millipore, USA). Recombinant lentiviruses were then concentrated by ultracentrifugation (2 h, 50,000 × *g*).

Lentivirus at the MOI of 30 was centrifuged (4℃, 1,200 × *g*) at for 1 h to infect Jurkat cell line. As well, lentivirus at the MOI of 5, supplemented with 4 µg/mL polybrene, was added to infect HER293T cell line for 72 h, respectively. ZsGreen-positive cells were sorted by flow cytometry (FACS Calibur III, Becton Dickinson) for coculture experiments.

### Flow cytometry

#### pNKs and NK92-CD16a cell activation

GP/sGP/ssGP in PBS (100 µg/mL, 100 µL/well) was coated overnight at 4°C in 96-well plates. Wells were washed three times with cold PBS and blocked with 20% BSA-PBS for 2 h at 37℃. mAbs (MIL77-3, MIL77-3F, Mab114, rEBOV548) were incubated at 20 µg/mL for an additional 1 h at 37°C. Unbound antibodies were removed by cold PBS. pNKs and NK92-CD16a cells were added at 3 × 10^5^ cells/well in the presence of 4 mg/mL brefeldin A (Sigma, Cat. No. B5936), 5 mg/mL GolgiStop (BD Biosciences, Cat. No. 554724), and PE anti-human CD107a antibody (Biolegend, Cat. No. 328608) for 5 h at 37°C. Uncoated protein and mAbs were added simultaneously, and the plates were incubated. Cells were fixed (Invitrogen, Cat. No. 00522356) and permeabilized (Biolegend, Cat. No. 421002) according to the manufacturer’s instructions and stained for PE anti-human IFN-γ antibody (Biolegend, Cat. No. 506507). The cells were then fixed with 4% paraformaldehyde and detected by flow cytometry (BD). The raw data were analyzed by FlowJo software.

#### Inhibition of NK cell activation

The inhibitors (wortmannin, MK-2206, rapamycin, PD98059) (Selleck, Cat. No. S2758, S1078, S1039, S1177) were dissolved in DMSO and pre-incubated with pNKs and NK92-CD16a cells at 37°C for 30 min. Cells were then co-incubated with uncoated sGP/mAbs complex for 5 h. The remaining steps were the same as above.

#### Activation of NK92-CD16a cells by beads-complex

Artificial beads (Dynabeads M-450 Epoxy, Invitrogen, 14013) were mixed with sGP (100 µg/mL) overnight at 4℃. MIL77-3 (20 µg/mL) was incubated with beads-sGP complex at 37°C for 1 h and added to stimulate NK92-CD16a cells. The remaining steps were the same as above.

#### Activation of NK92-CD16a cells by cell membrane-anchored complex

ZsGreen-positive Jurkat and HEK293T cell lines were sorted, respectively, and pre-incubated with 20 µg/mL MIL77-3 at 37°C for 1 h. These cell lines were then mixed with NK92-CD16a cells at the indicated ratio for 5 h. CD107a expression in CD56^+^ cells was detected as described above. sGP binds to pNKs: 5 × 10^5^ pNKs or NK92-CD16a cells were centrifuged to remove the supernatant and incubated with 100 µg/mL biotinylated-sGP (bio-sGP) for 30 min at 4°C. Cold PBS was used to wash unbound bio-sGP, and APC-conjugated anti-human CD3 (Biolegend, Cat. No. 304610), PE-conjugated anti-human CD56 (Biolegend, Cat. No. 304606), and FITC-conjugated avidin (Biolegend, Cat. No. 405101) were added and incubated for 30 min at 4°C.

#### Detection of CD16 expression in NK cells

5 × 10^5^ NK92-CD16a cells or pNKs were centrifuged to remove the supernatant and incubated with PE-labeled anti-human CD45 (BD Horizon, Cat. No. 564357), FITC-labeled anti-human CD3 (Biolegend, Cat. No. 300306), APC-labeled anti-human CD56 (Biolegend, Cat. No. 304610), PerCP-labeled anti-human CD16 (Biolegend, Cat. No. 302030) for 30 min at 4°C. Unbound antibody was washed out by cold PBS, and the cells were fixed with 4% paraformaldehyde. sGP/MIL77-3 complex administration *in vivo*

### sGP/MIL77-3 complex administration *in vivo*

#### The complex activates lymphocytes *in vivo*

C57BL/6J female mice (6–8 week) were purchased from Vital River Laboratories (Beijing, China). These mice were maintained in specific pathogen-free conditions. The experimental protocol was approved by the Animal Ethics Committee of the Beijing Institute of Pharmacology and Toxicology (IACUC-DWZX-2020-697). Mice were injected intraperitoneally with sGP/MIL77-3 or beads-coupled sGP/MIL77-3 complex (sGP: 100 µg/mL; MIL77-3: 20 µg/mL, 100 µL/mouse). Spleens were removed 12 h later, and lymphocytes were isolated using Ficoll gradient (TBD sciences, Cat. No. LTS1092). APC-labeled anti-mouse CD3 (Biolegend, Cat. No. 100236), FITC-labeled anti-mouse NK1.1 (Biolegend, Cat. No. 108706), PE-labeled anti-mouse CD69 (Biolegend, Cat. No. 104507), and PE-labeled anti-mouse CD25 (Biolegend, Cat. No. 101903) were incubated with lymphocytes at 4°C for 30 min. Unbound antibody was washed out by cold PBS, and the cells were fixed with 4% paraformaldehyde.

#### The complex binds to T cells

Non-activated and mouse T-activator CD3/CD28 (Thermo, Cat. No. 11452D)-activated lymphocytes were incubated with 50 µg/mL biotinylated sGP/control protein for 30 min at 4°C. FITC-conjugated avidin, PE-conjugated anti-mouse CD4 (Biolegend, Cat. No. 100408), and APC-conjugated anti-mouse CD8α (Biolegend, Cat. No. 100711) were then incubated with lymphocytes for 30 min. Cells were fixed by 4% paraformaldehyde and detected by flow cytometry.

### Statistical analysis

Data were analyzed, and the graphs were plotted using Prism software (GraphPad Prism 8). The data are presented as the mean ± standard error of the mean. Intergroup differences were compared using unpaired *t*-test or ANOVA. The *P* < 0.05 was considered statistically significant.

## RESULTS

### MIL77-3 binds to sGP and shares the epitopes with Mab114 and rEBOV548

**Fig 1:**
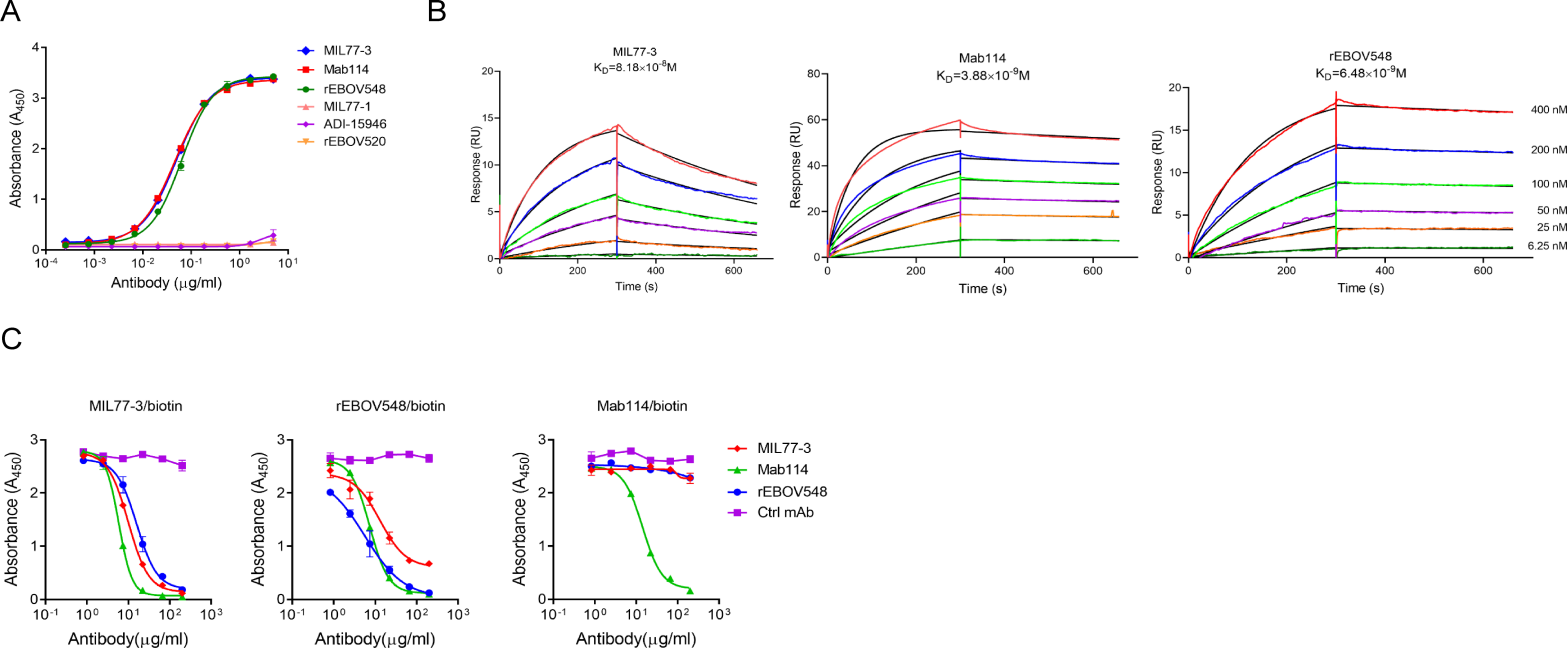
MIL77-3 engages sGP and shares epitopes recognized by Mab114 and rEBOV548. (**A, B**) The binding capacity of the indicated monoclonal antibodies to sGP was determined by ELISA (**A**) and SPR assays (**B**), respectively. (**C**) The competitive engagement of three indicated mAbs with sGP was detected by competition ELISA. The representative data from three independent experiments were shown.

Several therapeutic mAbs directed against the enveloped GP have been developed to combat EBOV (14). First, we determined the binding capacity of these mAbs to sGP. MIL77-3, Mab114, and rEBOV548 displayed high-affinity binding to sGP with similar binding activities, as shown by ELISA (Fig. 1A) and SPR (Fig. 1B), respectively. While, MIL77-1, ADI-15946, and rEBOV520 did not bind to sGP. This appears reasonable that the epitopes recognized by MIL77-3, Mab114, and rEBOV548 reside mainly or in part in the glycan cap region, which is included in the structure of sGP. Conversely, MIL77-1, ADI-15946, and rEBOV520 engage the core of GP1 (6, 15, 16), which is impeded by steric occlusion of glycan cap, leading to inaccessibility of these mAbs to sGP. Next, we compared the competitive binding capacity of these three mAbs. MIL77-3 and rEBOV548 competed with each other and Mab114 also antagonized the engagement of MIL77-3 or rEBOV548 with sGP. Whereas, MIL77-3 or rEBOV548 could not hinder the binding of Mab114 to sGP (Fig. 1C), indicating that the epitopes between MIL77-3, Mab114, and rEBOV548 are overlapped partially.

### MIL77-3/sGP complex augments the cytotoxicity of pNKs to lymphocytes

**Fig 2:**
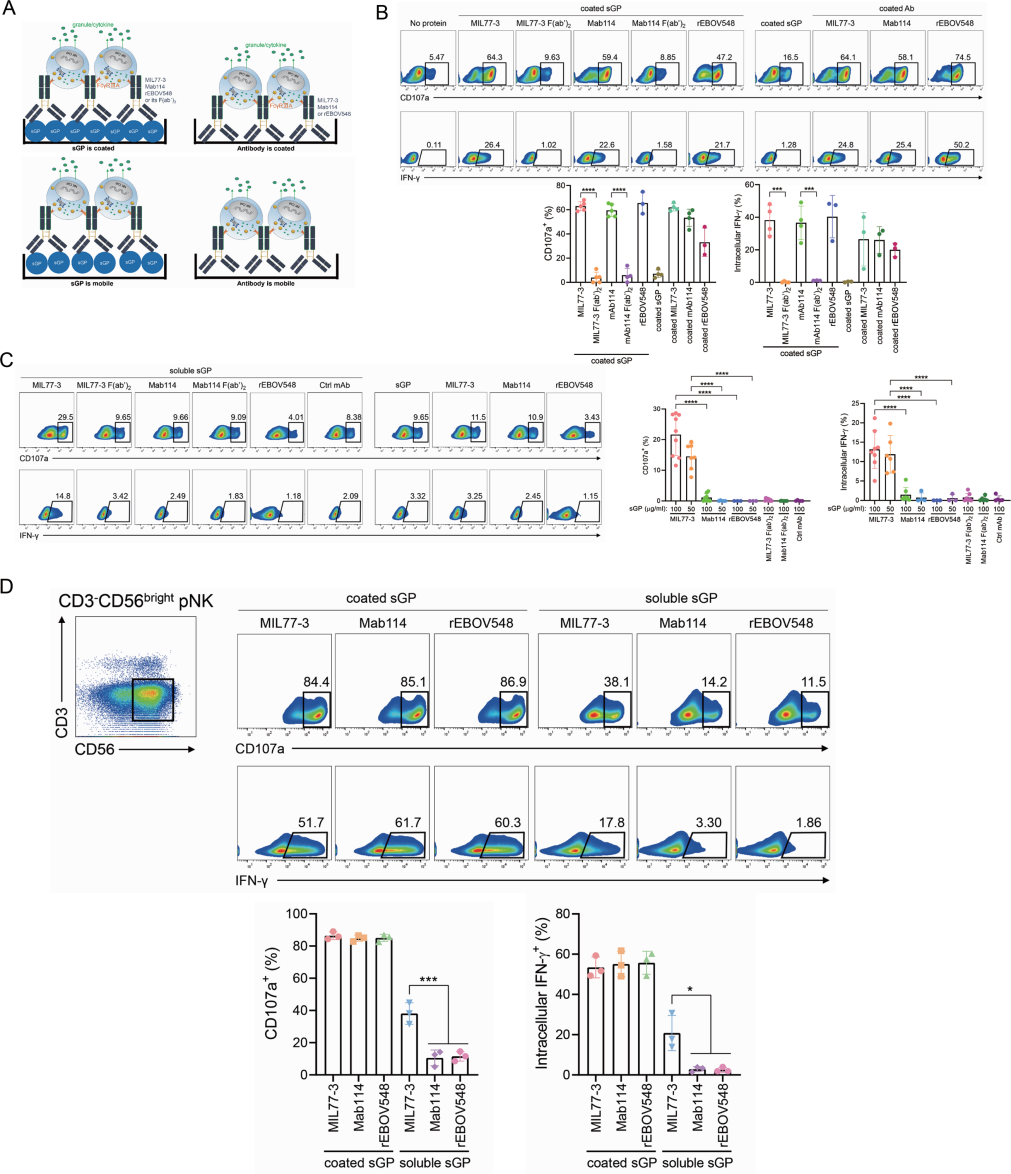
sGP/MIL77-3 complex has the ability to trigger pNK activation. (**A**) A scheme of the protocols on determination of the ability of coated or mobile sGP/mAb complex or mAb alone to stimulate NK cytotoxic activity. (**B**) sGP or mAbs were immobilized and engaged the indicated mAbs or F(ab’)_2_ fragments. pNKs were added and incubated for 5 h. (**C**) Soluble sGP alone or in combination with the indicated mAbs were incubated with pNKs for 5 h. (**D**) Immobile and soluble sGP in combination with the indicated mAbs were incubated with pNKs for 5 h, respectively. CD3^−^CD56^bright^ pNKs were gated. Cytotoxicity marker (CD107a, IFN-γ) expression was detected by flow cytometry. Representative plots were shown. The data were pooled from 3 to 8 healthy volunteers (*P < 0.05, ***P < 0.001, ****P < 0.0001).

It is well-known that MIL77-3, Mab114, and rEBOV548 have the potency to elicit the ADCC function of NK cells upon ligation of enveloped GP (6, 16, 17). We, thus, compared the ADCC-inducing ability of these three mAbs alone or in combination with sGP according to the protocol shown in Fig. 2A. Immobile sGP/MIL77-3 complex stimulation significantly increased the expression of degranulation marker (CD107a) and IFN-γ in pNKs and Fc deletion rendered the loss of NK activation (Fig. 2B; Fig. S2), indicating that NK-activating function of the complex is Fc-dependent. Furthermore, the similar effect was observed between MIL77-3, Mab114, and rEBOV548 (Fig. 2B), suggesting that although MIL77-3 rather than Mab114 and rEBOV548 is glycoengineered to augment ADCC function, Fc modification has minimal effects on ADCC-inducing ability of immobile sGP/mAb complex. Interestingly, coated mAbs alone also displayed a strong NK-activating function (Fig. 2B), which was equal to that of the sGP/mAb complex. While, coated sGP alone did not significantly elevate CD107a and IFN-γ expression of pNKs (Fig. 2B), leading to the conclusion that mAb instead of sGP is responsible for the ADCC-inducing ability of sGP/mAb complex.

To mimic the virtual context of the coexistence of sGP and mAbs, we detected the NK-activating capacity of mobile sGP, mAbs alone, or complex. In contrast to the immobile form, mobile sGP/Mab114 or rEBOV548 complex or mAbs alone did not increase CD107a and IFN-γ expression of pNKs (Fig. 2C). Importantly, mobile sGP/MIL77-3 complex retained NK-activating capacity although the abundance was relatively low compared with the immobile form (Fig. 2C). Intriguingly, the formation of the complex was required for its NK-activating ability, as mobile sGP or MIL77-3 alone was unable to elicit NK responses (Fig. 2C). Furthermore, we showed that ssGP also bound to MIL77-3 and that the complex of mobile ssGP/MIL77-3 had comparable potency to trigger NK activation (Fig. S3A and B). In sharp contrast, although GP protein without mucin-like domain bound to MIL77-3, uncoated GP/MIL77-3 complex had no effects on triggering NK degranulation (Fig. S3C and D).

It is noteworthy that pNKs contain at least three populations: CD3^−^CD56^bright^ pNKs, CD3^−^CD56^dim^ pNKs, CD3^+^CD56^−^ T lymphocytes. The virtual expression of CD16 on the surface of these populations was readily observed, albeit with varying magnitude (Fig. S4). Upon exposure to a stimulus, T cells could also be evoked to release cytotoxic granules and IFN-γ (18–20). It is, thus, possible that T cells in pNKs contribute to the effect of sGP/MIL77-3 complex described above. To address this, the three populations in pNKs were gated, respectively, and then CD107a as well as IFN-γ expression was detected. Consistent with the results shown in Fig. 2B and C, sGP/MIL77-3 complex stimulation led to significantly elevated expression of CD107a and IFN-γ by CD3^-^CD56^bright^ and CD3^−^CD56^dim^ pNKs (Fig. 2D; Fig. S5A). While, the expression levels of these two markers in T cells exposed to sGP/MIL77-3 were much lower than that in two NK subsets. Importantly, mobile sGP/MIL77-3 complex did not have more potency to trigger degranulation and IFN-γ expression in T cells compared with sGP/Mab114 or rEBOV548 (Fig. S5B). Therefore, NK cells, including CD56^bright^ and CD56^dim^ subpopulations in pNKs, play a dominant role in sGP/MIL77-3-induced cytotoxicity.

### PI3K/AKT/mTOR pathway is involved in sGP/MIL77-3 complex-elicited cytotoxicity

**Fig 3:**
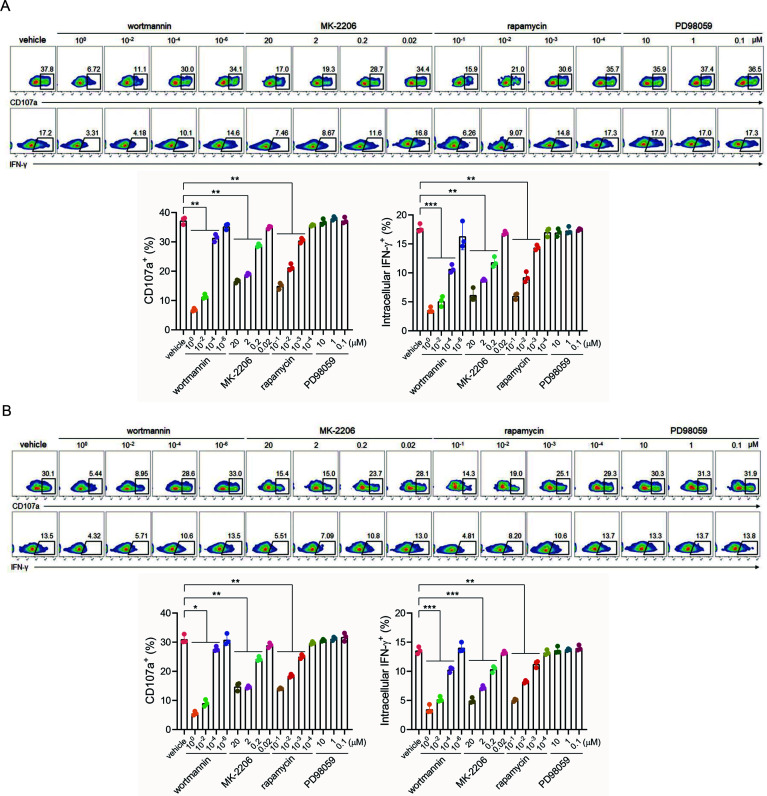
The ADCC-inducing effects of sGP/MIL77-3 complex are mediated by PI3K/AKT/mTOR signaling pathway. pNKs were pre-incubated with the indicated inhibitors for 30 min and then stimulated by soluble sGP/MIL77-3 complex for 5 h. CD3^−^CD56^bright^ (**A**) and CD3^−^CD56^dim^ (**B**) subsets were gated, respectively, and CD107a and IFN-γ expression was detected by flow cytometry. Upper panel: representative plots; lower panel: the data pooled from three healthy volunteers (* *P* < 0.05, **P < 0.01, ***P < 0.001).

PI3K/AKT/mTOR and MAPK/ERK signaling pathways have been reported to participate in IgG1-mediated induction of ADCC (21). Therefore, we determine the involvement of these pathways in sGP/MIL77-3 complex-induced NK activation by utilizing appropriate inhibitors. As expected, the addition of inhibitors to PI3K (wortmannin), AKT (MK-2206), or mTOR (rapamycin) remarkably reduced CD107a/IFN-γ expression in sGP/MIL77-3 complex-stimulated pNKs, including CD56^bright^ and CD56^dim^ subpopulations, in a dose-dependent manner (Fig. 3A and B). In contrast, MAPK/ERK inhibition (PD98059) had no inhibitory effects on NK activation (Fig. 3A and B).

### Fucosylated MIL77-3 loses the ability to elicit NK cytotoxicity

**Fig 4:**
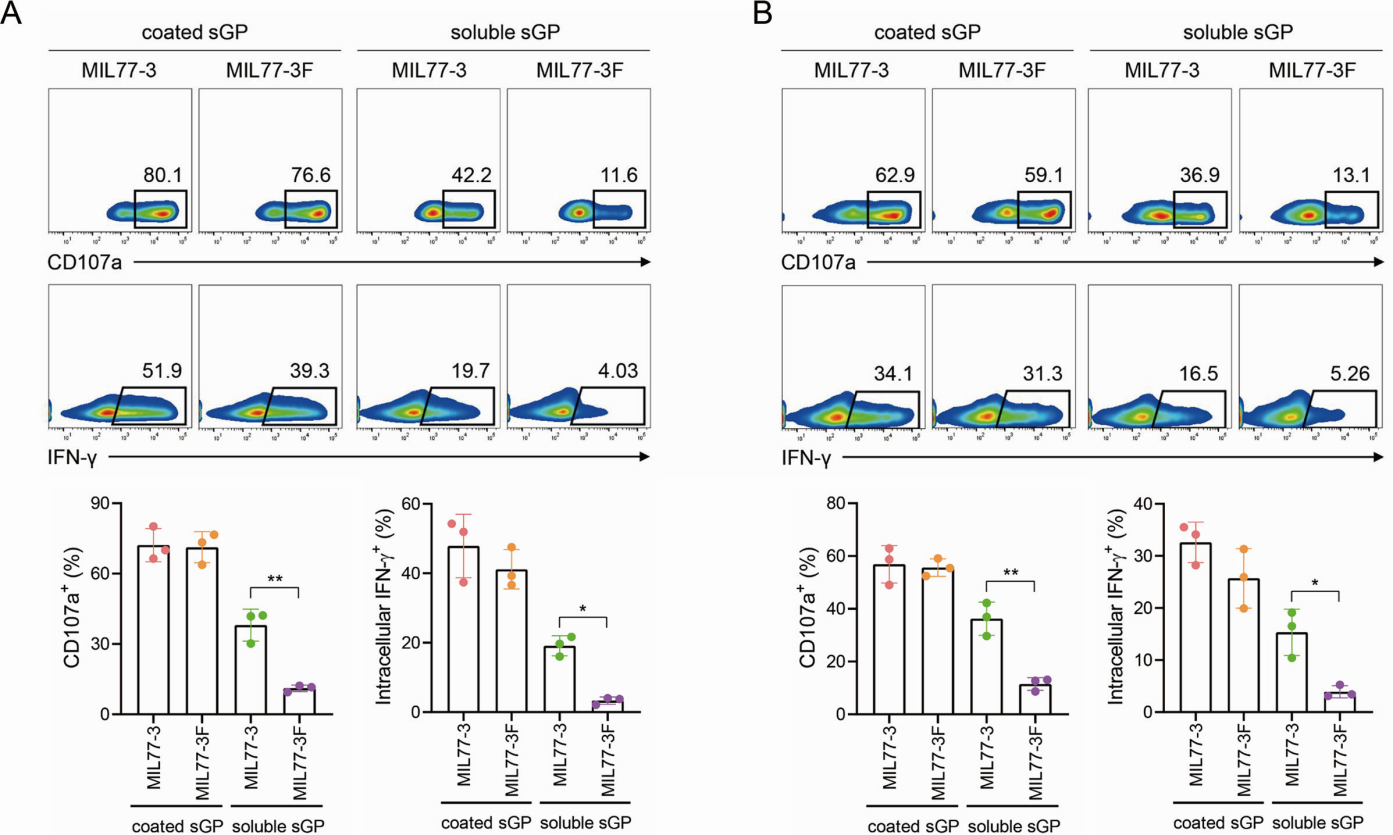
Fucosylated MIL77-3 engaged with sGP is unable to trigger pNK activation. Plated-coated or soluble sGP (100 µg/mL) engaged with MIL77-3 or MIL77-3F (20 µg/mL) and incubated with pNKs for 5 h. CD3^−^CD56^bright^ (**A**) and CD3^−^CD56^dim^ (**B**) subsets were gated, respectively, and CD107a and IFN-γ expression was detected by flow cytometry. Upper panel: representative plots; lower panel: the data pooled from three healthy volunteers (*P < 0.05, **P < 0.01).

To determine the role of MIL77-3 afucosylation in its potency to trigger NK activation, we produced fucosylated version of MIL77-3 termed as MIL77-3F (Fig. S6A through C) and compared the ability of these two mAbs to induce ADCC. Similar to Mab114/rEBOV548 with heavy fucosylation, coated sGP/MIL77-3F at high doses triggered CD107a/IFN-γ expression in CD56^bright^ and CD56^dim^ pNKs, respectively, which was comparable to that exposed to sGP/MIL77-3 (Fig. 4A and B). The pNK-stimulating function of low-dose sGP/MIL77-3F was dramatically impaired as expected, compared with the afucosylated counterparts (Fig. S7A and B). Importantly, the mixture of mobile sGP and MIL77-3F could not elicit NK degranulation as well as IFN-γ release (Fig. 4A and B), indicating that afucosylated Fc portion of MIL77-3 is indispensable for sGP/MIL77-3-mediated NK activation.

### Mobile sGP/MIL77-3 complex cannot elicit NK92-CD16a degranulation

**Fig 5:**
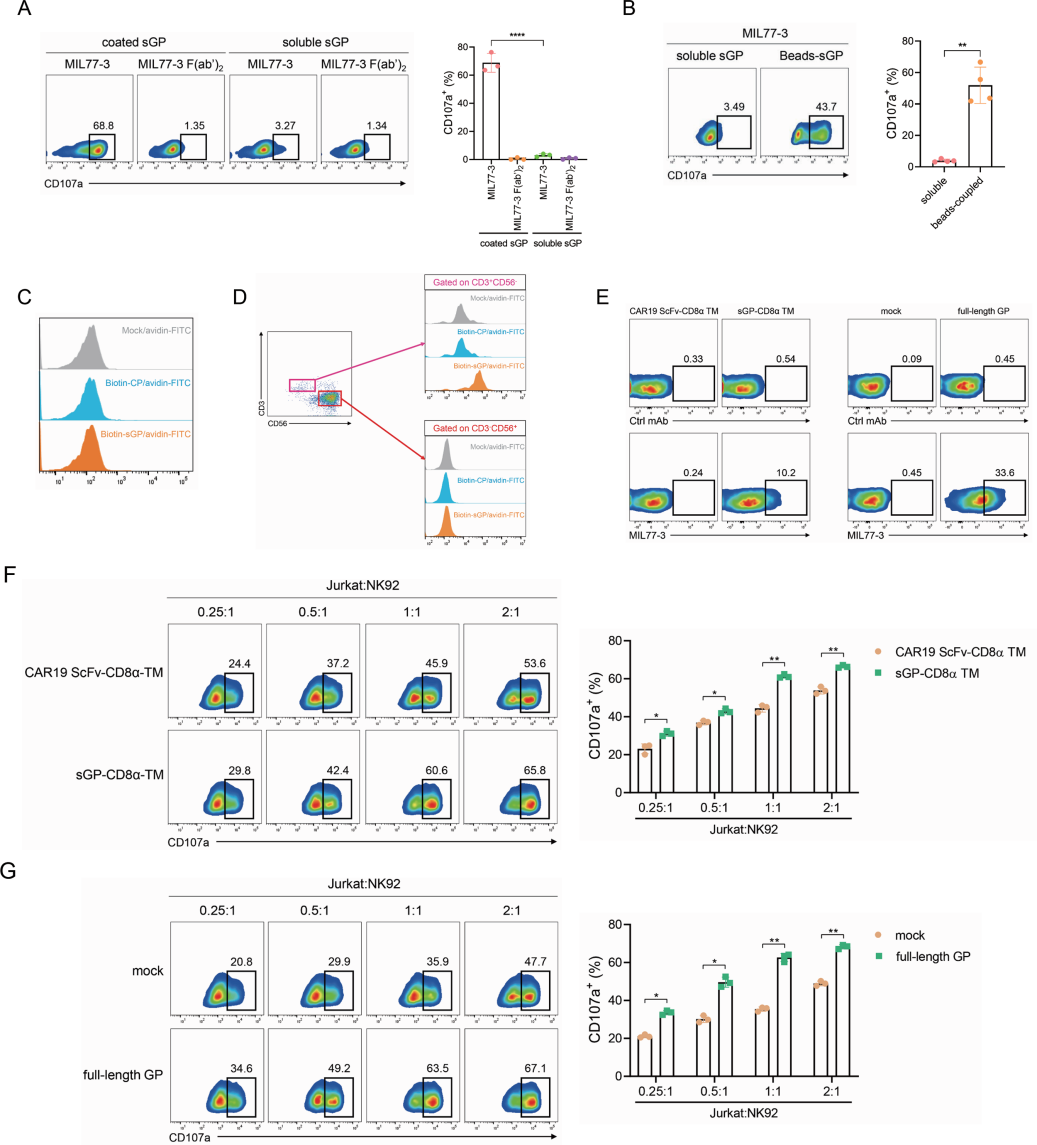
Soluble sGP/MIL77-3 complex is unable to trigger NK92-CD16a activation. (**A**) Plated-coated or soluble sGP engaged MIL77-3 or F(ab’)_2_ fragments and incubated with NK92-CD16a for 5 h. (**B**) Soluble or microbeads-conjugated sGP engaged MIL77-3 and incubated with NK92-CD16a for 5 h. CD107a expression was detected by flow cytometry. (**C**) NK92-CD16a were incubated with biotinylated sGP and FITC-conjugated avidin. The Fluorescence was detected by flow cytometry. (**D**) pNKs were gated on CD3^−^CD56^+^ and CD3^+^CD56^−^ populations, respectively. The binding capacity of sGP to these two populations was detected by flow cytometry. (**E**) Jurkat cells were infected with lentivirus loading sGP-CD8α TM, full-length GP, or control constructs. The expression of sGP or GP on the cellular membrane was detected by the combination of MIL77-3 and PE-conjugated anti-human IgG Fc antibody. (**F, G**) Jurkat cells expressing membrane-bound sGP (**F**) or GP (**G**) were co-cultured with NK92-CD16a at the indicated ratios in the presence of MIL77-3 (20 µg/mL) for 5 h. CD56^+^ cells were gated, and CD107a expression was detected by flow cytometry. Representative plots were shown. The data were pooled from three independent experiments (*P < 0.05, **P < 0.01).

We further determine the effects of sGP/MIL77-3 complex on NK activation using a NK cell line, which was stably transfected with CD16a and, thus, suitable to evaluate mAb-mediated NK activation (Fig. S8). Unexpectedly, mobile sGP/MIL77-3 did not induce CD107a expression in NK92-CD16a although immobile counterpart did so (Fig. 5A). This suggests that crosslinking of sGP may be requisite for NK-activating capacity of mobile sGP/MIL77-3. To address this issue, we utilized microbeads to couple sGP and detected the ability of the complex of beads-coupled sGP and MIL77-3 to induce NK activation. Beads coupling, indeed, dramatically restored NK-activating capacity of mobile sGP/MIL77-3 (Fig. 5B). As expected, sGP did not bind to the membrane of NK92-CD16a (Fig. 5C). Considering that slight contamination of other immune cell populations in pNKs is inevitable during cell processing, we assumed that sGP was tethered to the membrane of these immune cells. Similar to NK cell line, sGP also did not bind to pNKs (Fig. 5D). Conversely, the engagement of sGP with T lymphocytes (CD3^+^CD56^−^) was visible obviously (Fig. 5D), indicating that mobile sGP/MIL77-3 triggers pNK degranulation via tethering sGP to an unrecognized receptor on the surface of T cells instead of NK population. To further address this issue, sGP and GP were displayed on the surface of a human T cell line (Jurkat) via introducing tandem sGP-CD8α TM region construct and full-length GP sequences, respectively. Membrane-bound sGP or GP could be recognized by MIL77-3 smoothly (Fig. 5E). sGP or GP-decorated T cell line in combination with MIL77-3 was, thus, leveraged to activate NK92-CD16a cells. Consequently, Jurkat cells loading membrane-anchored sGP or GP plus MIL77-3 led to significantly increased expression of CD107a in NK92-CD16a cells, compared with those introduced by CAR19-ScFv-CD8α TM or control construct in combination with MIL77-3 (Fig. 5F and G). Intriguingly, the infection of lentivirus containing sGP-CD8α TM or full-length GP to a human epithelial cell line (HEK293T) also rendered the augmented degranulation of NK92-CD16a upon the addition of MIL77-3 (Fig. S9A through C). Overall, these data clearly indicate that the engagement of sGP with an unknown receptor in T cells is required for the NK-stimulating function of sGP/MIL77-3 complex.

### The complex of beads-coupled sGP/MIL77-3 activates NK cells *in vivo*

**Fig 6:**
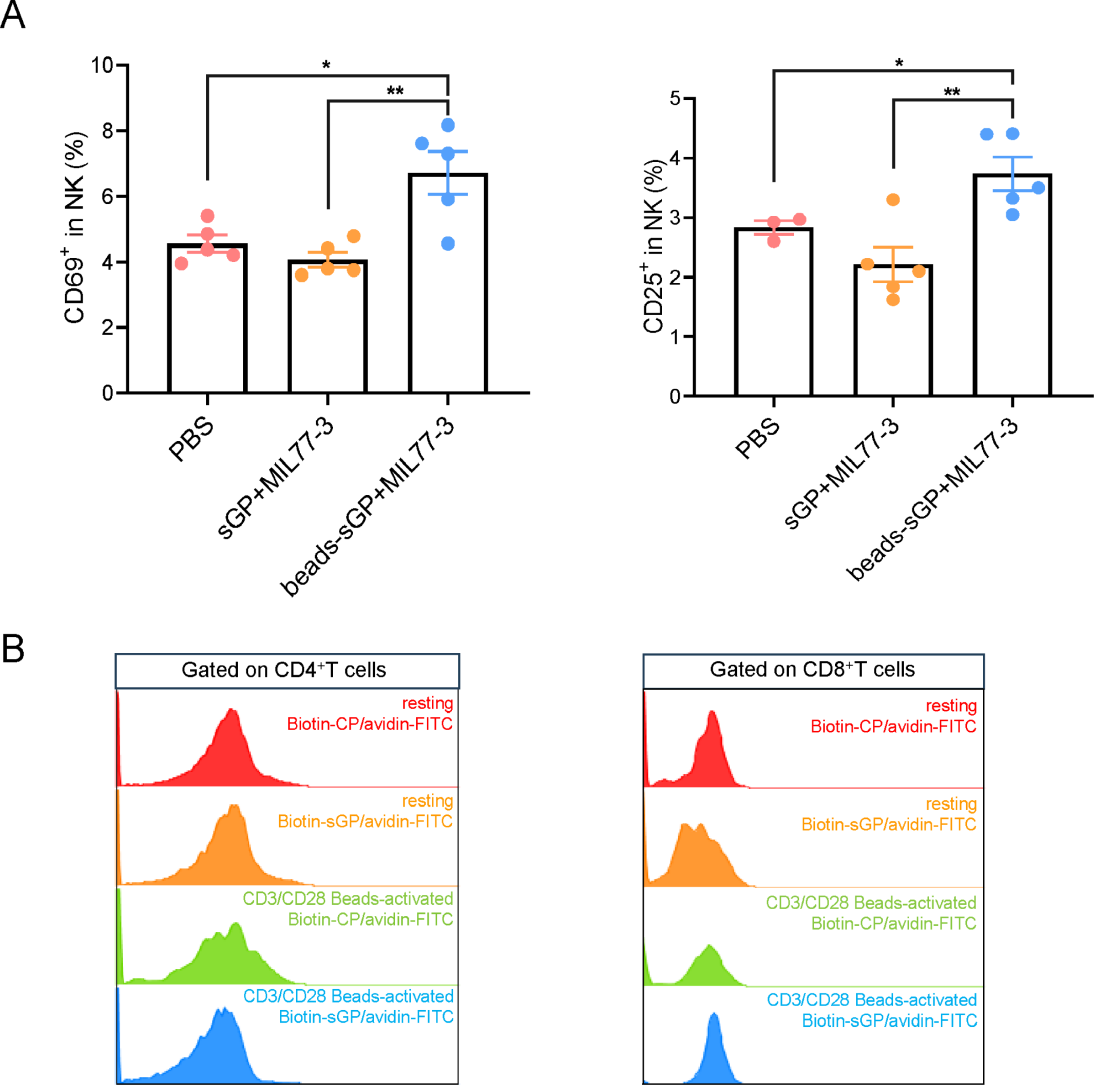
Beads-coupled sGP/MIL77-3 complex administration activates murine NK cells *in vivo*. (**A**) Beads-coupled or native sGP/MIL77-3 complex was injected intraperitoneally into C57BL/6 mice. Twelve hours later, PBMCs in the spleen were isolated and CD69 as well as CD25 expression in CD3^−^NK1.1^+^ cells was detected by flow cytometry. (**B**) The binding of sGP to resting and CD3/CD28 microbead-activated murine T cells was detected by flow cytometry. (*P < 0.05, **P < 0.01).

Next, we determined the effects of sGP and MIL77-3 on NK activation *in vivo*. Injection of the mixture of beads-coupled sGP and MIL77-3 elicited the significant up-regulation of activation markers (i.e., CD69 and CD25) expression in murine NK cells (Fig. 6A). Notably, sGP/MIL77-3 complex administration did not lead to NK cell activation (Fig. 6A). Furthermore, sGP was not attached to the cellular membrane of resting or primed murine T lymphocytes (Fig. 6B), which can account for unresponsiveness of NK to sGP/MIL77-3 complex *in vivo*.

## DISCUSSION

**Fig 7:**
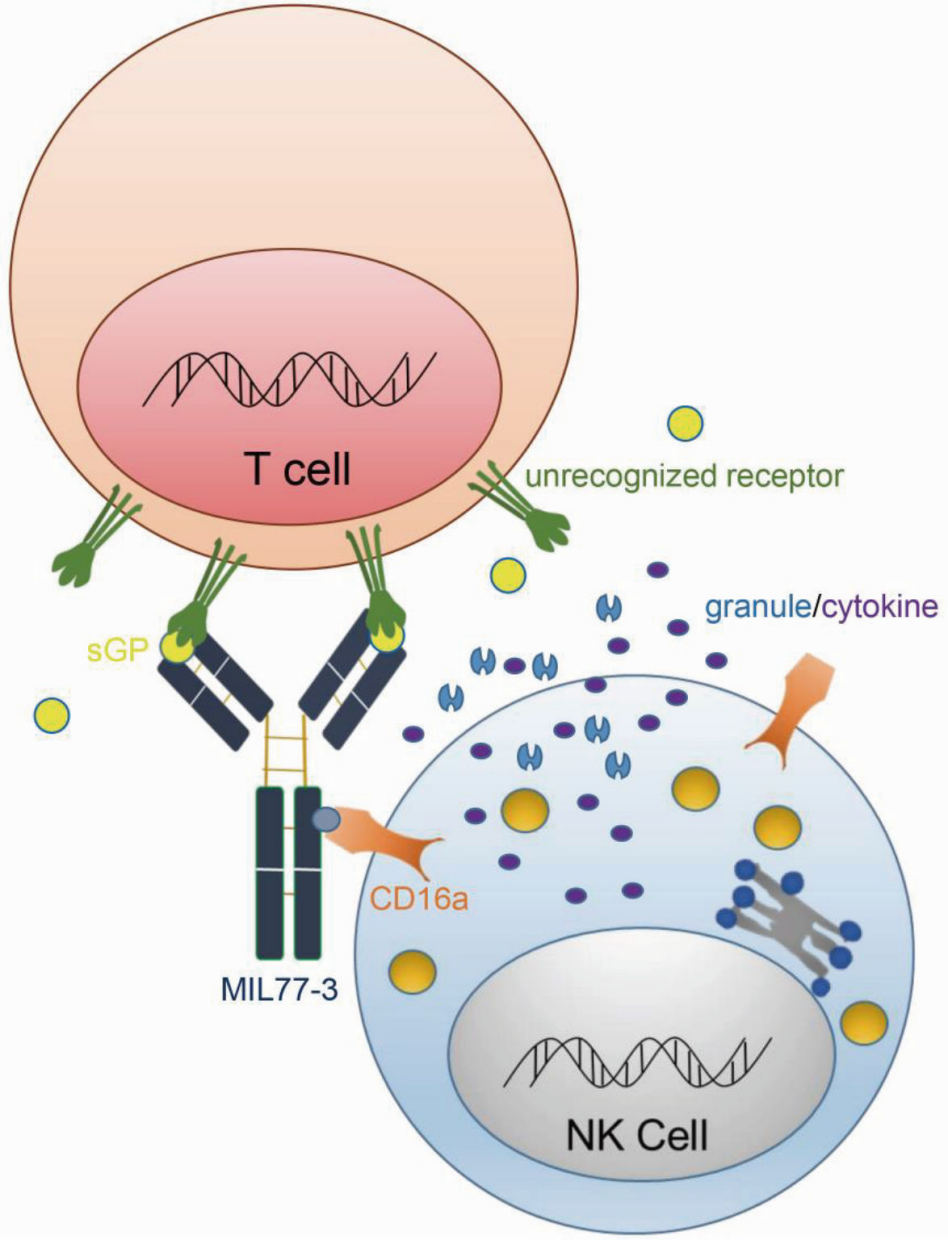
The schematic illustration on the mode of action of sGP/MIL77-3 complex. MIL77-3 potently binds to sGP, a glycoprotein secreted by ebolavirus, to form the immunocomplex. sGP is crosslinked to an unrecognized receptor on the surface of immune cells (e.g., T cells). Consequently, the complex robustly triggers NK degranulation and release of pro-inflammatory cytokines via the interaction between the afucosylated Fc of MIL77-3 and CD16a on the surface of NK cells.

MIL77-3 is a therapeutic mAb with Fc modification to enhance ADCC function that recognizes the glycan cap of GP1 and clinical trials on its efficacy against EBOV infection is ongoing. The potential impact of this modification on host immune responses, however, remains elusive. In this study, we demonstrate for the first time that MIL77-3 binds to sGP to form immunocomplex and thereby trigger the cytotoxicity of circulating NK cells (Fig. 7). The consequence of NK activation, however, is still unclear. Given the massive apoptosis of lymphocytes in EBOV-insulted hosts (22, 23), we assume that sGP/MIL77-3 complex-triggered NK cytotoxicity may lead to lymphocyte death. The co-culture of pNKs and T cell lines or primary T cells in the presence of sGP and MIL77-3 did not render T cell death. Furthermore, sGP did not engage the membrane of T cell lines or primary T cells (data not shown). Therefore, further investigations on the outcome of sGP/MIL77-3-triggered NK activation are warranted.

Herein, we provide evidence that the ADCC-inducing ability of sGP/MIL77-3 complex is dependent on afucosylated Fc and membrane-anchored sGP. It is well established that fucose deletion in the constant region of mAb increases the binding affinity for FcγRIIIa and enhances ADCC function (24). On the other hand, the clustering of mAb/antigen complex is essential to form immune synapse to elicit NK cytotoxicity (25, 26). The binding of mAb alone or dissociated mAb/antigen complex to FcγRIIIa is, thus, not sufficient to switch on signaling cascade to trigger ADCC function. Similar to membrane-bound antigen, the binding of sGP to the receptor(s) on the surface of immune cells (e.g., T cells) or other cell populations renders the clustering of sGP/MIL77-3 complex to readily activate NK cells. Unfortunately, the receptor for sGP remains enigmatic although several molecules are reported to be the ligand for GP (27–29). Initially, FcγRIIIb was shown to be a receptor for sGP that impedes the physical linkage between FcγRIIIb and CR3 on neutrophil upon engagement (30). However, it was shown later that the binding observed was the result of the formation of an immune complex between FcγRIIIb and the Fc portion of the anti-sGP antibody (31). It is noteworthy that we did not detect the association between sGP and resting T cell line or primary blood-borne T lymphocytes (data not shown). Considering that T cells contaminated in pNKs might be at the state of activation in the presence of exogenous IL-2, sGP-recognized receptor(s) is likely induced to be expressed or translocated onto the cell surface upon activation. The virtual identity of receptor(s) warrants further investigation in the future.

One definitive role of sGP in the pathogenesis of EBOV infection is to dampen anti-GP immune responses by absorbing endogenous anti-GP antibodies with cross-reactivity to sGP (32). This resultant sGP/mAb complex unlikely leads to NK activation as we showed that sGP/mAb without Fc modification is unable to elicit cytotoxicity. Of note, the efficacy of therapeutic mAbs against EBOV challenge *in vivo* is tightly linked to the ability to activate NK cells, supporting glycomodification of Fc region of anti-EBOV mAbs as a putative strategy to enhance Fc-mediated immune effector function as well as protection *in vivo* (14, 33). The data in this work, however, highlight the potential harmful influence of this modification on host immune responses, which may impair the efficacy of mAbs. Therefore, it is imperative to evaluate the NK-stimulating capacity of afucosylated mAb engaged with sGP when a new candidate is developed. In addition, the development of mAbs recognizing the epitopes at the interface of sGP and its receptor engagement may be advantageous to retain the mobility of sGP by steric hindrance to the interaction between sGP and its receptor.

## Data Availability

The original contributions presented in the study are included in the article and supplemental material. Further inquiries can be directed to the corresponding authors.

## SUPPLEMENTAL MATERIAL

Supplemental material jvi.00685-24-s0001.docx Fig. S1 to S9.

ASM does not own the copyrights to Supplemental Material that may be linked to, or accessed through, an article. The authors have granted ASM a non-exclusive, world-wide license to publish the Supplemental Material files. Please contact the corresponding author directly for reuse.
