# Supplementary material for: Afucosylated anti-EBOV antibody MIL77-3 engages sGP to elicit NK cytotoxicity
Source: J Virol. 2024 Aug 20;98(9):e00685-24. doi: 10.1128/jvi.00685-24 (PMC11406966; doi:10.1128/jvi.00685-24)

**Supplementary Figure legends**

**Fig. s1. The purity of pNKs.**

The dead cells were excluded by 7-AAD staining. The NK cells were gated on CD45^+^CD3^-^CD56^+^. Representative plots were shown.

**Fig. s2. Removal of Fc segment does not affect the binding of MIL77-3/Mab114 to sGP.**

MIL77-3 and Mab114 F(ab’)_2_ were prepared as described in Methods. The binding capacity of intact MIL77-3/Mab114 as well as its F(ab’)_2_ version to sGP was determined by ELISA. Representative results from two independent experiments were shown.

**Fig. s3. MIL77-3 engaged with ssGP instead of GP triggers pNK activation.**

(A) The binding of MIL77-3 to ssGP was determined by ELISA. (B) Soluble ssGP engaged MIL77-3 or F(ab’)_2_ fragment and incubated with pNKs for 5 h. CD107a and IFN-γ expression was detected by flow cytometry. (C) The binding of MIL77-3 to GP was determined by ELISA. (D) Uncoated GP engaged MIL77-3 or F(ab’)_2_ fragment and incubated with pNKs for 5 h. CD107a and IFN-γ expression was detected by flow cytometry. Representative results from three independent experiments were shown.

**Fig. s4. Three cell populations in pNKs virtually express CD16.**

CD3^-^CD56^bright^, CD3^-^CD56^dim^, CD3^+^CD56^-^ subpopulations in pNKs were gated respectively and CD16 expression was determined by flow cytometry. Representative plots were shown.

**Fig. s5. The stimulatory function of sGP/MIL77-3 is observed in CD3^-^CD56^dim^ NKs but not in CD3^+^CD56^-^ T cells.**

Immobile and soluble sGP in combination with the indicated mAbs were incubated with pNKs for 5 h respectively. CD3^-^CD56^dim^ pNKs (A) and CD3^+^CD56^-^ T cells (B) were gated respectively. Cytotoxicity marker (CD107a, IFN-γ) expression was detected by flow cytometry. Representative plots were shown. The data were pooled from 3 healthy volunteers (* *p* < 0.05, ns: no significance).

**Fig. s6. Characterization of MIL77-3F.**

(A) The purity of MIL77-3F was determined by SDS-PAGE. (B) The binding of MIL77-3F to sGP was determined by ELISA. (C) HILIC analysis of the PNGase F enzymatically released N-glycans from MIL77-3 and MIL77-3F as well as Mab114 and rEBOV548 respectively. Representative results from two independent experiments were shown.

**Fig. s7. Fucosylated MIL77-3 engages with coated sGP to trigger pNK activation is impaired.**

Plated-coated sGP (100 μg/ml) engaged with MIL77-3 or MIL77-3F (2, 0.2, 0.02 μg/ml) and incubated with pNKs for 5 h. CD3^-^CD56^bright^ (A) and CD3^-^CD56^dim^ pNKs (B) were gated respectively. CD107a and IFN-γ expression was detected by flow cytometry. The representative plots from three independent experiments were shown.

**Fig. s8. NK92 expresses CD16a determined by flow cytometry.**

**Fig. s9. sGP or GP-expressing HEK293T cells in combination with MIL77-3 enable NK cytotoxicity.**

(A) HEK293T cells were infected with lentivirus loading sGP-CD8α TM, full-length GP or control constructs. The expression of sGP or GP on the cellular membrane was detected by the combination of MIL77-3 and PE-conjugated anti-human IgG Fc antibody. (B,C) HEK293T cells expressing sGP (B) or GP (C) were co-cultured with NK92-CD16a at the indicated ratios in the presence of MIL77-3 (20 µg/ml) for 5 h. CD56^+^ cells were gated and CD107a expression was detected by flow cytometry. Representative plots were shown. The data were pooled from three independent experiments (* *p* < 0.05, ** *p* < 0.01).

**Fig.s1**


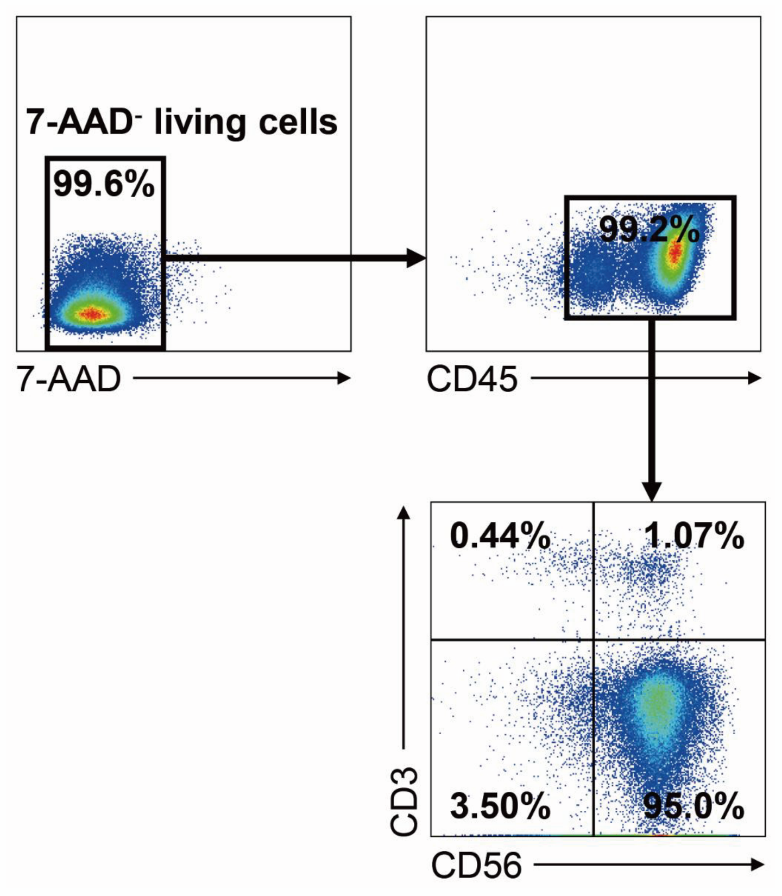


**Fig.s2**

**
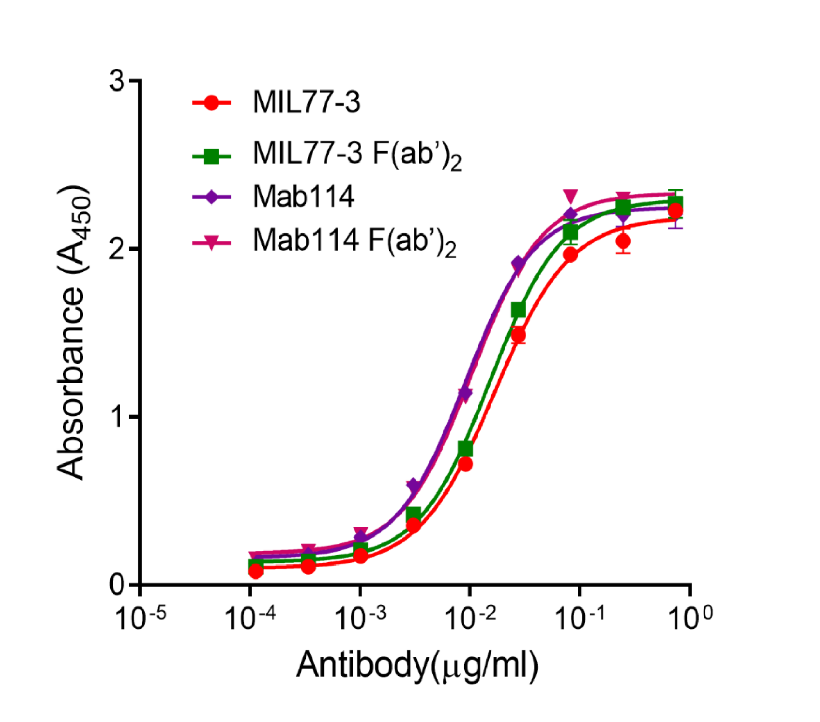
**

**Fig.s3**


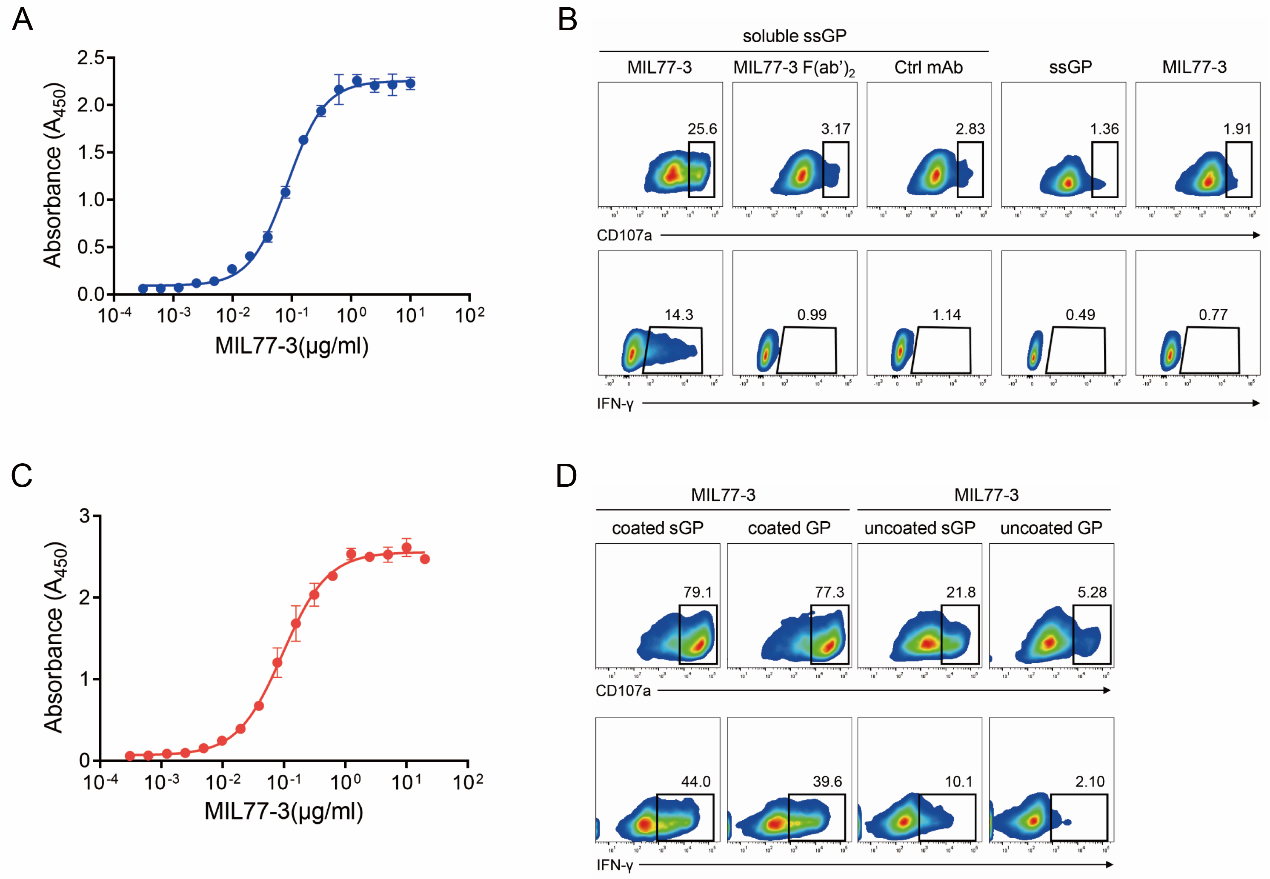


**Fig.S4**


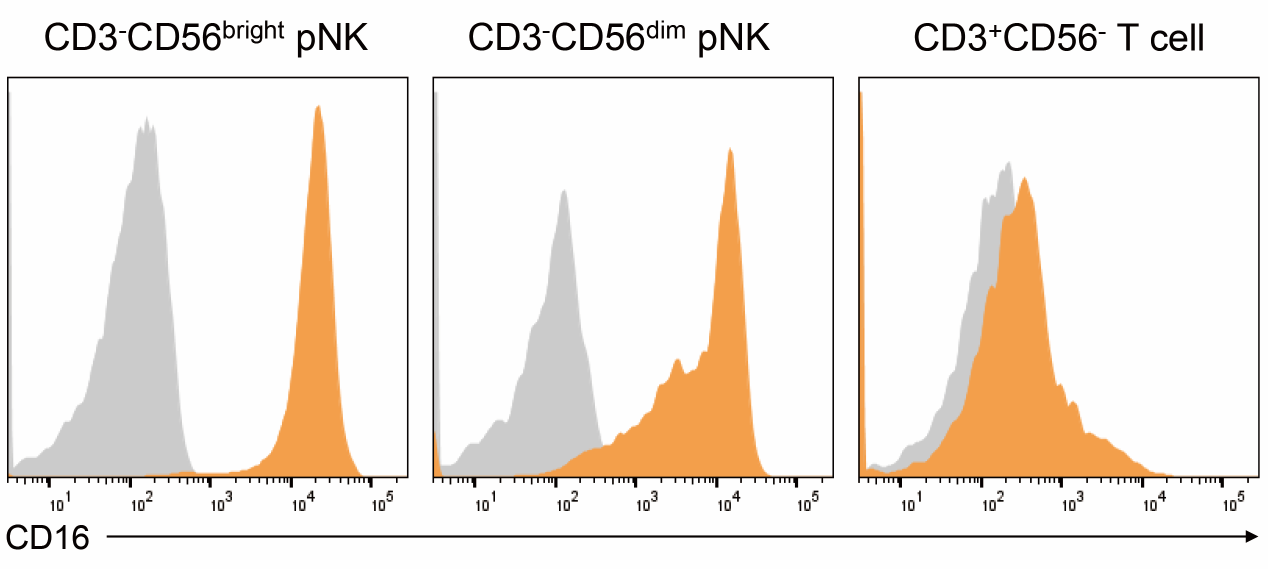


**Fig.s5**


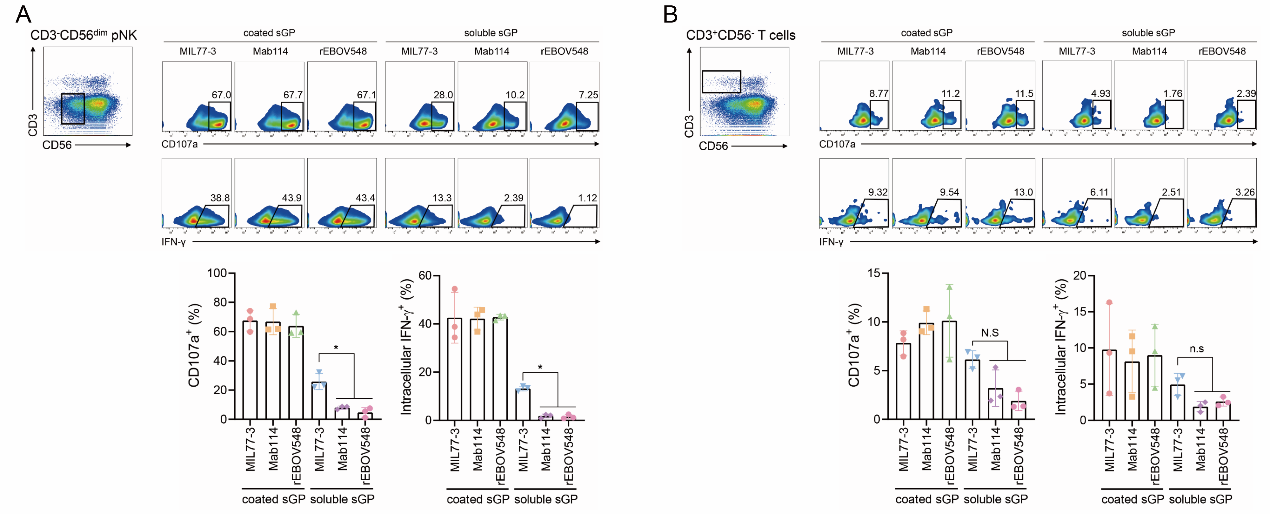


**Fig.s6**


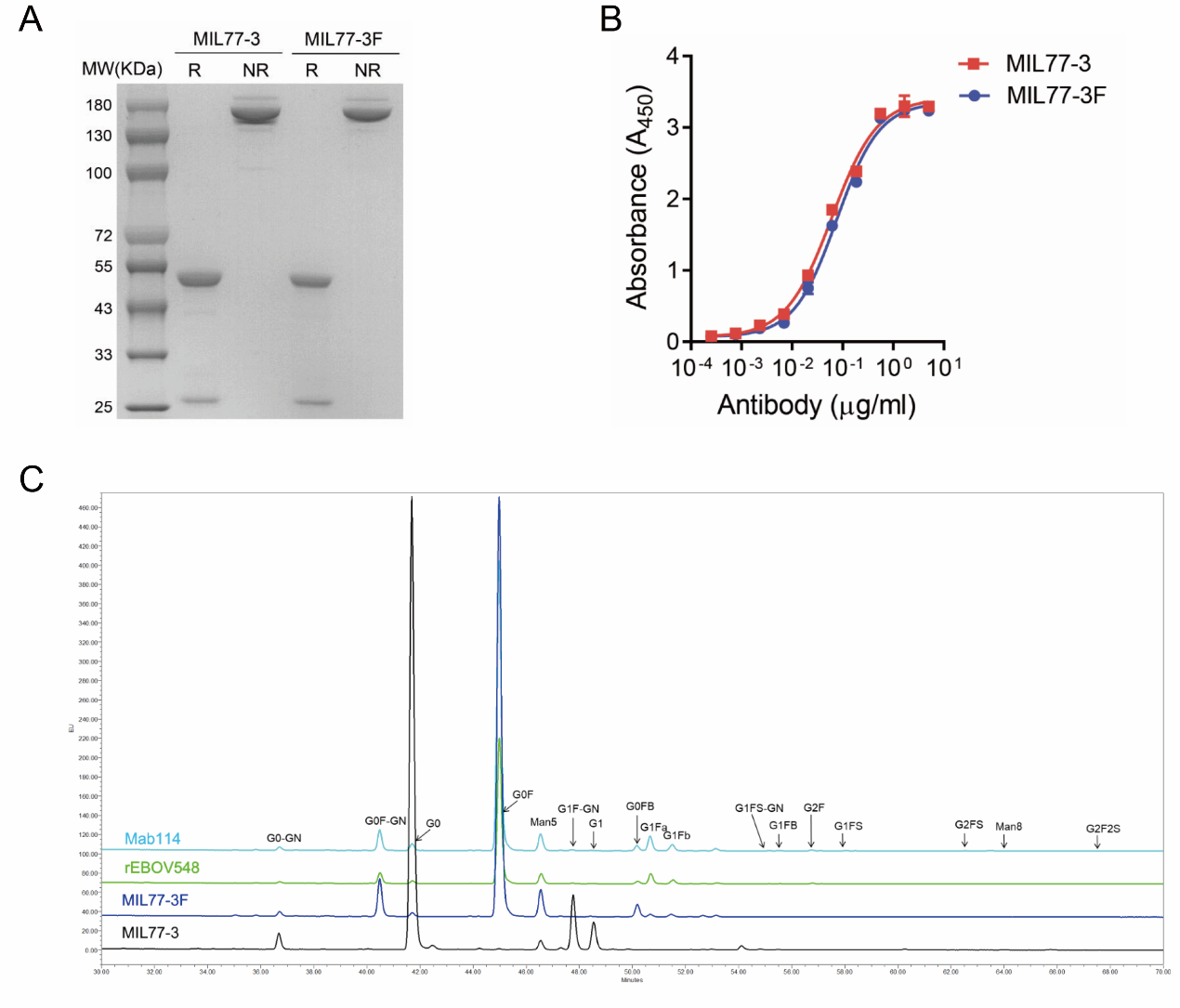


**Fig.S7**


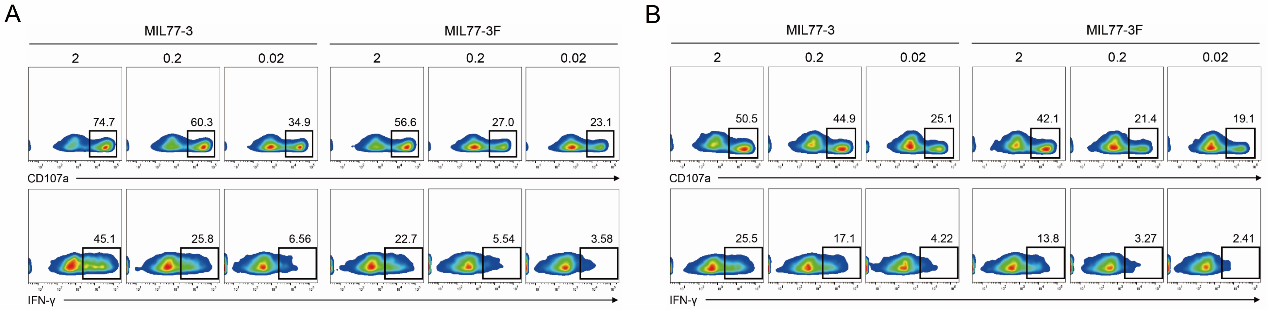


**Fig.S8**

**
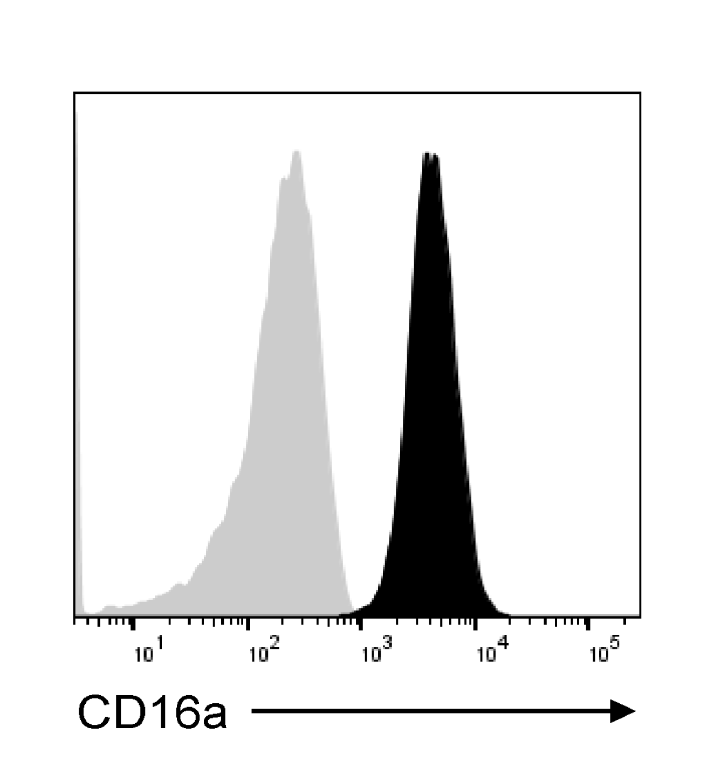
**

**Fig.s9**


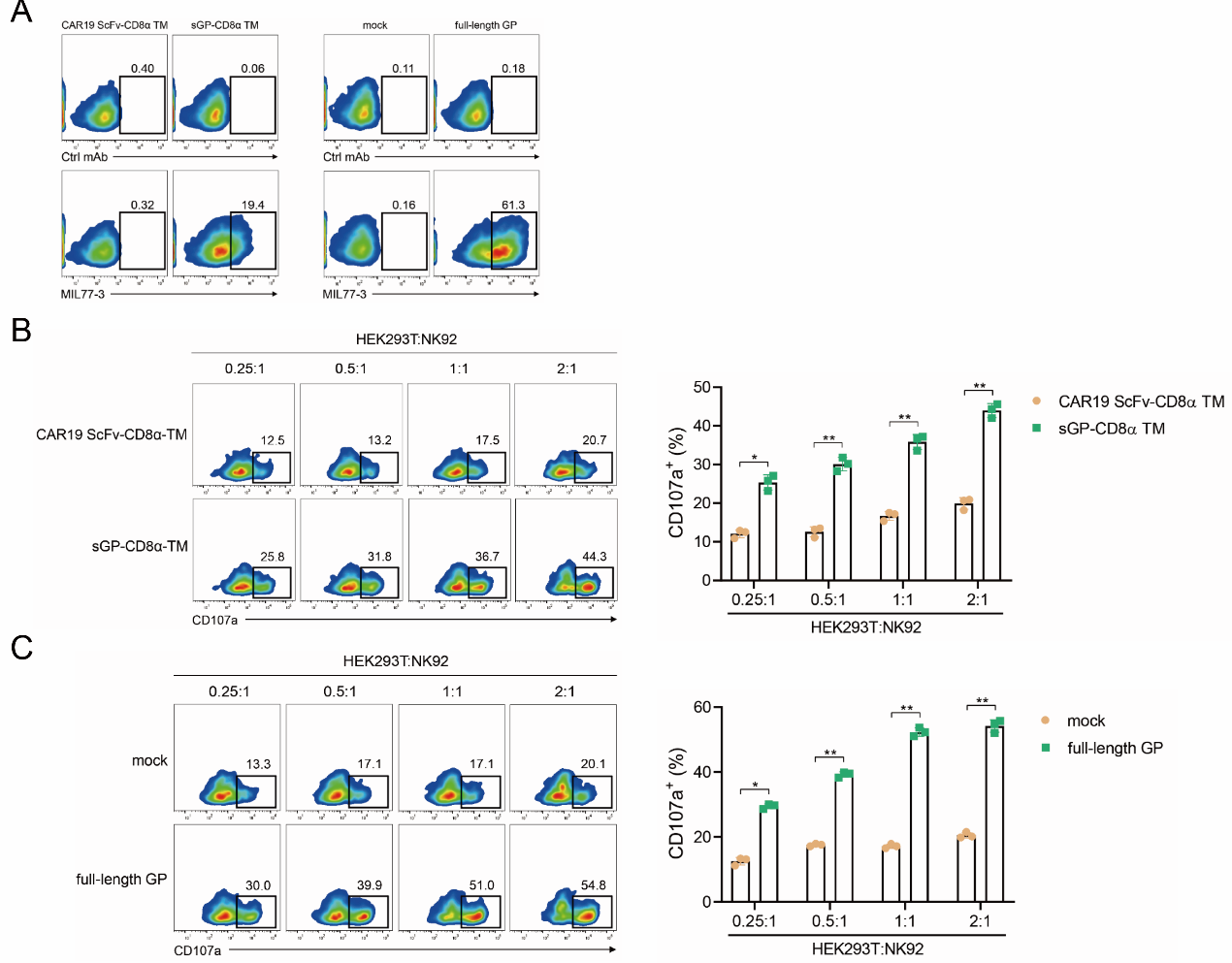

Supplement: Supplemental material — Fig. S1 to S9. [file jvi.00685-24-s0001.docx]
